# Oral delivery of carrier-free dual-drug nanocrystal self-assembled microspheres improved NAD^+^ bioavailability and attenuated cardiac ischemia/reperfusion injury in mice

**DOI:** 10.1080/10717544.2021.1886198

**Published:** 2021-02-19

**Authors:** Hongfei Nie, Yarong Zhang, Haiyang Yu, Hong Xiao, Tao Li, Qian Yang

**Affiliations:** a Laboratory of Mitochondrial and Metabolism, Department of Anesthesiology, National Clinical Research Center for Geriatrics, West China Hospital of Sichuan University, Chengdu, China; b Laboratory of Anesthesia and Critical Care Medicine, West China Hospital of Sichuan University, Chengdu, China; c State Key Laboratory of Oral Disease, National Clinical Research Center for Oral Diseases, West China Hospital of Stomatology, Sichuan University, Chengdu, China; d Laboratory of Plastic Surgery and Burns, State Key Laboratory of Biotherapy and Cancer Center, West China Hospital of Sichuan University, Chengdu, P. R. China

**Keywords:** Carrier-free microspheres, nicotinamide riboside, oral administration, NAD + Bioavailability, cardiac I/R injury

## Abstract

Nicotinamide riboside (NR), as a dietary supplement, can be converted to nicotinamide adenine dinucleotide (NAD^+^) in cells to support mitochondrial energy metabolism. However, the efficacy of oral administrated NR is limited due to its quick degradation in circulation and low bioavailability in targeted organs. In this study, we fabricated nanocrystal self-assembled microspheres by Nano Spray Dryer for oral delivery of NR. The structure of NR and resveratrol (RES) nanocrystal self-assembled microspheres (NR/RESms) is confirmed by the morphology, chemical structure, and crystallization. The NR/RESms displayed restricted NR release at the gastric acid-mimic condition (<15% in the first 8 hours), while achieved accelerated NR release in an enteric-mimic environment (>46% within 8 hours). Oral administration of NR/RESms for 8 hours significantly elevated NAD^+^ levels in serum (169.88 nM versus 30.93 nM in the NR group, *p* < .01; and 66.89 nM in the NR + RES group, *p* < .05), and enhanced NAD^+^ abundance in multiple organs in mice, exhibiting an improved oral NAD^+^ bioavailability. In addition, without any serious adverse effects on major organs, oral delivery of NR/RESms attenuated myocardial infarction (15.82% versus 19.38% in the I/R + NR group and 20.76% in the I/R + NR + RES group) in a cardiac ischemia/reperfusion (I/R) injury mouse model. Therefore, our data supported that the NR/RESms is a promising candidate as NAD^+^ booster for oral administration.

## 1. Introduction

Nicotinamide adenine dinucleotide (NAD^+^) is a critical redox coenzyme in cellular metabolism, participating in a variety of biochemical reactions, such as gluconeogenesis, ketogenesis, lipogenesis and detoxification of reactive oxygen species (Garten et al., 2015). As a major electron carrier for oxidative phosphorylation, it presents in both oxidized and reduced forms (NAD^+^/NADH) in mitochondria. The distribution of NAD^+^ and NADH in the mitochondria extremely depends on the balance of substrate metabolism and the oxidative phosphorylation that consumes NADH (Wang et al., 2016). NAD^+^/NADH homeostasis maintains the mitochondrial energy metabolism by tight coupling of tricarboxylic acid (TCA) cycle flux and the rate of oxidative phosphorylation, perturbation of which has been implicated in numerous diseases, including aging, cancer, heart failure, Alzheimer, diabetes, and obesity (Chiarugi et al., 2012; Yang et al., 2014; Lee et al., 2016; Rajman et al., 2018).

Nicotinamide riboside (NR) is a pyridine nucleoside form of vitamin B3, which naturally exists in milk and is a candidate of dietary supplement (Trammell et al., 2016; Martens et al., 2018). Normally, NR is converted to nicotinamide mononucleotide (NMN) by the biocatalysis of nicotinamide riboside kinases (NRK1,2), and subsequently converted to NAD^+^ by nicotinamide mononucleotide adenylyltransferase (NMNAT), serving as a NAD^+^ precursor (Trammell et al., 2016). Oral supplementation with NR molecules has been proven to increase the NAD^+^ level in heart, liver and skeletal muscle, as well as oxidative metabolism in high-fat diet-induced obesity (Cantó et al., 2012; Khan et al., 2014; Diguet et al., 2018; Schöndorf et al., 2018; Elhassan et al., 2019). However, oral administrated NR is quickly degraded to nicotinamide (NAM) in the gastrointestinal tract and liver, and is undetectable in blood stream within 1 hour (Liu et al., 2018). Thus, prolonging NAD^+^ bioavailability is vital in NR-based therapy.

The major challenge for oral administrated drug is to prevent undesired degradation of active ingredients in gastrointestinal environment (Viswanathan et al., 2017). Ideal carriers for oral drug delivery should provide sufficient protection and facilitate active ingredients to reach the targets (Griffin et al., 2016; Sosnik & Augustine, 2016; Li et al., 2019). In this regard, polymer materials are appealing carrier candidates (Sim et al., 2016; Qi et al., 2019; Yin et al., 2020). Their biocompatibility and controllable degradation properties endow a controlled release of drugs in gastrointestinal tract environment (Ribeiro et al., 2017; Yang et al., 2019). Moreover, their adhesion property would also extend the retention time (Brown et al., 2020). However, the low drug-loading capacity, poor physical stability, and difficulty in industrial manufacturing of polymer materials hinder their applications (Sheikhpour et al., 2017).

Unlike polymers-based micro- or nano-dispersion systems, carrier-free drug-assembled system has some unique characteristics, including high drug-loading capacity and precisely controlled morphology. Among these carrier-free drug delivery systems, nanocrystal is a novel formulation approach to increase the solubility of hydrophobic drugs and control drug release rate (Lu et al., 2019). Small molecules nanocrystals can be self-formed by hydrophilic and hydrophobic drugs without pharmaceutic adjuvant, exhibiting a great potential for industrialization (Fontana et al., 2018; Mohammad et al., 2019). In our previous studies, hydrophobic small molecular drugs have been introduced to construct a nanoassembly with hydrophilic near-infrared (NIR) dye (Peng et al., 2019, 2020). The results indicated that hydrogen bond and hydrophobic driving force among the hydrophilic and hydrophobic drugs facilitate the self-assembly of nanocrystals and yield tunable particle size and morphology (Yang et al., 2020).

The natural polyphenol resveratrol (RES) serves as an excellent small molecular self-assembled drug (Koushki et al., 2018; Prysyazhna et al., 2019). More importantly, it has been reported to increase NMNAT activity and enhance NAD^+^ biosynthesis (Grant, 2010; Mouchiroud et al., 2013; Mathieu et al., 2016). However, the oral bioavailability of RES is limited due to its poor solubility and rapid metabolism in the body (Neves et al., 2012；Novelle et al., 2015). If combination of NR and RES as dual-drug nanocrystals, it would hold promise to enhance the therapeutical potential of both drugs.

The nanoscale particle size and large specific surface area of drug nanocrystals result in high surface energy, which become unstable in solution (Yang et al., 2018; Liu et al., 2019). By spray drying process, the nanocrystals can be assembled to form an aggregated structure with improved physical stability (Sverdlov Arzi & Sosnik, 2018). The nanocrystal self-assembly is not only conducive to storage and pharmaceutic preparation, but also prolongs the retention of drugs in the gastrointestinal tract (PA Ferreira et al., 2020).

**Scheme 1.**
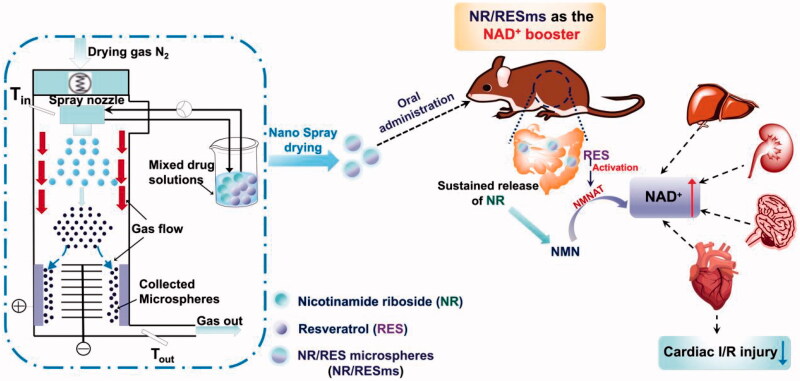
The NR/RES-assembled microspheres are constructed by the Nano Spray Dryer B90 to improve oral bioavailability of nicotinamide riboside (NR). The sustained release of NR in an enteric environment endowed the microspheres for oral administration with significant increase of NAD^+^ levels in circulation and multiple organs, which offered myocardial protective effect in a mouse cardiac ischemia/reperfusion injury model.

In the present study, we fabricated carrier-free NR/RES nanocrystal self-assembled microspheres (NR/RESms; Scheme 1) and suspended in natural polysaccharides sodium alginate solution for oral administration. The optimal NR:RES ratio and particle morphology, as well as the physicochemical parameters of NR/RESms were investigated. The efficacy in NAD^+^ elevation was evaluated by the NAD^+^ levels in serum and multiple organs after oral delivery. The histopathological changes in major organs, and the protective effect on cardiac ischemia/reperfusion (I/R) injury were also examined.

## 2. Materials and methods

### 2.1. Materials

Nicotinamide riboside (NR, Mw: 255.25 g/mol) was obtained from Yuanye Biotechnology, Co. Ltd (Shanghai, China). Resveratrol (RES, Mw: 228.25 g/mol) was obtained from Meilun Biotechnology, Co. Ltd (Dalian, China). Sodium alginate, Sodium 1-heptanesulfonate monohydrate, Ethanol and Methanol were purchased from Aladdin Co. Ltd (Shanghai, China). NAD^+^/NADH-Glo™ Assay Kit was obtained from Promega Co. Ltd (USA). Triphenyltetrazolium chloride (TTC) was purchased from Sigma-Aldrich Co. Ltd (Saint Louis, USA).

### 2.2. Preparation of NR/RESms

The NR/RESms were formulated by Nano Spray Dryer B-90 (Buchi Corporation, New Castle) with spray cap of 5.5 μm. The atomizer air flow rate was 110 mL/min. The feeding ratio of NR and RES, the composition of solvent and the inlet temperature were considered as the process parameters to be evaluated. Meanwhile, outlet air temperature for each batch was recorded. The dried NR/RESms were collected by the electrostatic particle collector, and the yield of microspheres was calculated. For *in vivo* oral administration, the NR/RESms were suspended in sodium alginate solution.

### 2.3. Characterization of NR/RESms

#### Morphology

The micromorphology of NR/RESms was observed by scanning electron microscope (SEM, Hitachi SU3500, SU8220, Japan) and transmission electron microscope (TEM, Joel JEM-2100F, Japan).

#### Size distribution

The size distribution and surface charge of NR/RESms were measured by dynamic light scattering (DLS, Malvern Masterzizer 2000, UK).

#### X-ray diffraction (XRD)

Crystallographic assays for NR powder, RES powder, physical mixture of NR and RES, and NR/RESms were performed by X-ray Diffractometer (Panalytical X'PERT PRO), with the X-Ray source of Cu Kα. The data were collected by step-scan mode from 5° to 60° with a scan speed of 0.083°s^−1^.

#### Fourier transform infrared spectroscopy (FT-IR)

The chemical characteristic of the pure NR powder, pure RES powder, and NR/RESms was detected by a FT-IR spectrometer (Nicolet 6700, Thermo Scientific, USA). Transmission infrared spectra were obtained within the wavenumber range of 400-4000 cm^−1^.

#### Differential scanning calorimetry (DSC)

Thermal analysis for pure NR, pure RES, and NR/RESms powders were conducted by the DSC1 instrument (Mettler-Toledo, Switzerland). The thermograms for different samples were acquired at nitrogen atmosphere from room temperature to about 300 °C with a speed of 10 °C min^−1^.

#### Thermogravimetric (TG) analysis

The TG analysis for NR/RESms was performed using a Mettler-Toledo TGA2 instrument in nitrogen atmosphere. For analysis, NR/RESms powder was heated from 30 to 330 °C with a scanning rate of 10 °C min^−1^.

### 2.4. Drug loading and release profiles of NR/RESms in vitro

The amount of NR in NR/RESms was quantified by high-performance liquid chromatography (HPLC, Agilent 1260, USA) with a GU-C18M column (4.6 mm × 250 mm, 5 µm). The mobile phase was methanol-0.1% sodium heptanesulfonate buffer (20/80, v/v), pH = 3.5. The column temperature was set at 30 °C, and the detection wavelength was 261 nm. The drug-loading (DL) for NR was calculated according to the following formulas: DL (%) = (W_NR_/W_NR/RESms_) × 100.

The drug releasing experiments were performed in 25 mL 0.1 M hydrochloric acid solution (HCl, pH 1.2) or phosphate buffer solution (pH 6.8) to mimic the conditions of stomach and intestines *in vitro*. The NR and NR/RESms in alginate dispersions (5 mgNR/ml) were placed in pre-activated dialysis bag with molecular weight cutoff of 8 − 14 kDa, and rotated at 50 rpm in releasing medium at 37 °C with a water bath shaker. The releasing medium samples were collected at 0,0.5, 1, 2, 4, 8, 12, 24, 48, and 72 hours after experiment. The alternative release experiment was conducted by placing the NR/RESms in 25 mL 0.1 M HCl (pH 1.2) for 24 hours and then switching to phosphate buffer solution (pH 6.8) for another 24 hours. The releasing medium samples were collected at 0, 0.5, 1, 2, 4, 8, 12, 24, 28, 32, 36, and 48 hours after experiment. The NR amounts in the withdrawn samples were quantified by HPLC as mentioned above.

### 2.5. Pharmacokinetic analysis of NR/RESms in mice

All animal experiments were performed with the approval of the Institutional Animal Care and Use Committee of the West China Hospital, Sichuan University, China. The animals received humane care in compliance with the *Guide for the Care and Use of Laboratory Animals* published by the US National Institutes of Health. Male C57BL/6 mice, 8 to 10 weeks old, were purchased from Dashuo Experimental Animal Center, China. Mice were maintained in a vivarium with a 12-hour light/dark cycle at 22 °C and free access to food and water.

To analyze the impacts on NAD^+^ bioavailability, mice were treated by gavage of vehicle, NR, NR + RES mixture (weight ratio 1:3), or NR/RESms at a dosage of 100mgNR/kg. Before gavage, NR, NR + RES or NR/RESms was dispersed in sodium alginate solution to a working concentration of 50 μg NR/μl. Mice in the vehicle group received sodium alginate solution alone. The mice were sacrificed at 4 and 8 hours after gavage by cervical dislocation, and blood, heart, brain, kidney and liver samples were harvested. The NAD^+^ levels and NAD^+^/NADH ratio in plasma and tissue samples were measured using a NAD^+^/NADH-Glo™ Assay Kit (#G9072; Promega, USA).

### 2.6. Histopathological analysis after NR/RESms administration

The heart, brain, kidney and liver samples were fixed with 10% neutral buffered formalin, paraffin embedded and cut into 5-μm-thick sections. The sections were deparaffinized and rehydrated by immersing in xylene and alcohol. For hematoxylin and eosin (H&E) staining, the sections were stained with hematoxylin for 8 min and eosin for 1 min, washed in 0.5% hydrochloric acid alcohol, dehydrated and mounted. All sections were observed through a microscope (AX10 imager A2/AX10 cam HRC, Carl Zeiss, Germany) and photographed using ZEN software (Zeiss). For each section, ten to fifteen images were acquired from randomly selected fields. The representative image for each group was selected to illustrate the average of the group based upon the histological feature.

### 2.7. Mouse model of cardiac ischemia/reperfusion (I/R) injury

Myocardial I/R injury was induced by ligation of left anterior descending coronary artery (LAD) for 30 minutes and reperfusion for 24 hours as previously described (Li et al., 2017). Briefly, mice were anesthetized by intraperitoneal injection of ketamine (120 mg/kg) and xylazine (4 mg/kg), intubated and ventilated with pure O_2_, using a MiniVent mouse ventilator (Model 845, Harvard Apparatus). Body temperature was kept at 35–36 °C with a temperature-controlled surgical table. After a left thoracotomy was performed through the fifth intercostal space, the LAD was ligated with a 6-0 silk suture for 30 minutes of ischemia. At the end of ischemia period, reperfusion was accomplished by releasing the slipknot and the chest was closed in layers. After 24 hours of reperfusion, transthoracic echocardiography was performed, and the mice were sacrificed for infarct size measurement. In sham-operated mice, the silk suture placed underneath the LAD artery was not ligated. Four hours after gavage of vehicle, NR, NR + RES or NR/RESms, the mice were randomly assigned to the Sham, I/R + Vehicle, I/R + NR, I/R + NR + RES and I/R + NR/RESms groups.

### 2.8. Transthoracic echocardiography

Transthoracic echocardiography was performed on mice using a 13.0 MHz linear probe (Vivid 7; GE Medical System) and interpreted by an echocardiographer blinded to the experimental design. M-mode images were obtained from a parasternal short-axis view at the mid-ventricular level with a clear view of papillary muscle. Ejection fraction (EF) of the left ventricle was measured from the M-mode image under a heart rate around 550 bpm.

### 2.9. Infarct size assessment

I/R induced myocardial infarction was determined by Evans blue/TTC double staining as we described previously (Li et al., 2017). Briefly, after 24 hours of reperfusion, the ligature around the LAD was retied and the heart was quickly excised and mounted on a Langendorff apparatus. After washing out the blood by saline, 0.2 ml 1% Evans blue dye was injected through aortic root. The hearts were sliced transversally into 1-mm-thick sections and 5–6 sections per heart starting from the apex. The sections were incubated in 1% TTC in PBS at 37 °C for 15 minutes and then fixed in a 4% paraformaldehyde solution for 24 hours at room temperature. The area at risk (AAR) was identified by the absence of blue dye. The total LV area, AAR and TTC-negative staining area (infarcted myocardium) were measured with Image J software (NIH). Myocardial infarction was expressed as a percentage of the infarct size (IS) over AAR (IS/AAR) and the size of AAR was expressed as the percentage of AAR over total LV area.

### 2.10. Statistical analysis

Statistical analysis was performed using GraphPad Prism v.8.0.1 (GraphPad, La Jolla, CA). Values are presented as mean ± SD, and one-way ANOVA followed by Tukey's multiple comparisons test was performed to determine differences among groups. P-values <0.05 were considered statistically significant.

## 3. Results and discussion

### 3.1. Process optimization of NR/RESms

The drug ratios and process parameters of spray drying process might affect the properties of obtained products (Arpagaus et al., 2018). The NR/RESms were formulated by Nano Spray Dryer with the air flow rate of 110 mL/min according to our preliminary experiment. The process parameters, such as the weight ratio of two components, inlet temperature and outlet air temperature were investigated, and the influence on the yield, drug content, particle size, polydispersity index and zeta potential of different batches were evaluated. All the results were listed in the Tables 1 and 2.

**Table 1.** Optimization of microspheres in this study.

Batch code	NR (%w/w)	RES (%w/w)	Outlet air temperature(^o^C)	Yield (%)	Particle size distribution*				Zeta potential
					D*_10_* (μm)	D*_50_* (μm)	D*_90_* (μm)	Span	
1	50	50	33	NA	NA	NA	NA	NA	NA
2	40	60	33	9.66 ± 1.5	6.77 ± 0.025	40.3 ± 0.300	125.7 ± 6.49	2.95 ± 0.168	+32.00 ± 0.25
3	33	67	35	20.74 ± 2.7	3.53 ± 0.287	19.6 ± 2.05	62.7 ± 0.325	3.04 ± 0.322	+18.03 ± 0.55
4	25	75	38	62.40 ± 4.2	0.687 ± 0.039	2.59 ± 0.186	7.98 ± 1.33	2.80 ± 0.296	+4.36 ± 0.11
5	20	80	39	59.10 ± 3.9	0.219 ± 0.017	1.02 ± 0.251	6.21 ± 0.809	5.99 ± 0.659	+6.37 ± 0.09

Data are presented as the mean ± SD (*n* = 3).

**Table 2.** The conditions of nano spray-drying, yield of products and NR content of each batch.

Batch code	EtOH/H_2_O (v/v)	Drying air flow (L/min)	Inlet temperature(^o^C)	Outlet air temperature(^o^C)	Yield (%)	NR Content (% w/w)
4-1	1:2.5	110	105	NA	NA	NA
4-2	1:1.5	110	95	42	74.6 ± 3.4	22.3 ± 1.35
4-3	1:1	110	80	38	62.40 ± 4.2	22.0 ± 4.11
4-4	1.5:1	110	80	35	34.2 ± 2.5	22.7 ± 3.80
4-5	2:1	110	80	NA	NA	NA

Data are presented as the mean ± SD (*n* = 3).

**Figure 1.**
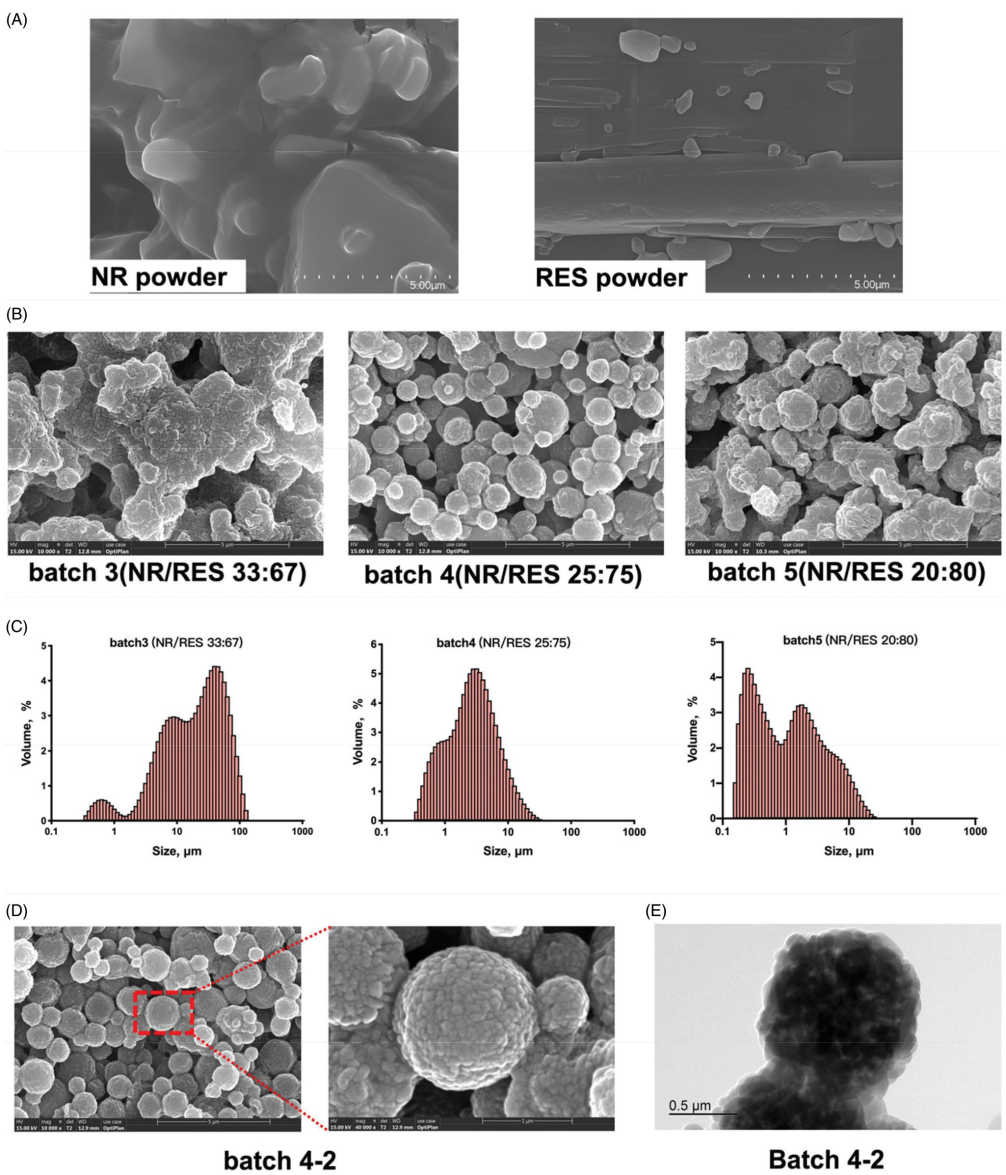
Characterization: (A) The micromorphology of NR and RES powders; SEM imaging (B) and DLS analysis (C) of NR/RES formulations with different drug weight ratio; the SEM imaging (D) and TEM imaging (E) of the optimized NR/RESms formulation (NR/RESms, batch 4-2).

The hydrophilic and hydrophobic components ratio displayed notable impacts on the product yield, and the properties of the obtained microspheres. The particle sizes for the NR/RESms in different batches were within the range of 1.02 ± 0.251 to 40.3 ± 0.300 μm, with the span of 2.80 ± 0.296 to 5.99 ± 0.659 (Table 1). The surface charges of the NR/RESms formulations in batch 1 to 4 were +32.00 ± 0.25, +18.03 ± 0.55, +4.36 ± 0.11, and +6.37 ± 0.09 mV, respectively. These results show that the cationic surface charge were decreased by reducing the amount of NR, presumably due to the ionization of NR molecule. SEM photographs for pure NR, and spray-drying powder (batch 2-4) were showed in Figure 1(A). With the increased feeding ratio of hydrophilic component NR, bigger and more aggregative microspheres were acquired (batch 2 and 3). When the proportion of NR reached 20%-25%, the obtained microspheres displayed superior homogeneity and dispersibility, which indicated that the crystallinity of hydrophobic components contributes to the particle formation and is benefit to the reduction of particle size distribution (Figure 1(A,B)). Meanwhile, the optimal yield for microspheres was achieved when NR feeding proportion reached 25%.

Based on the above analysis, the feeding ratio for NR and RES used in the following experiment was set according to the batch 4, and more precise adjustments were listed in Table 2. Higher yields and drug contents were achieved with an appropriate composition of solvent (1:1.5 v/v of EtOH/H_2_O) and an inlet temperature of 95 °C. The spherical and wrinkled surfaces were observed in the spray-dried microspheres (batch 4-2), which was formed by stacking crystalline particles (Figure 1(C)). The raspberry-like surface morphology was further observed by the TEM imaging (Figure 1(D)), suggesting the nanocrystal self-assembled structure of microspheres.

### 3.2. Characterization of NR/RESms

**Figure 2.**
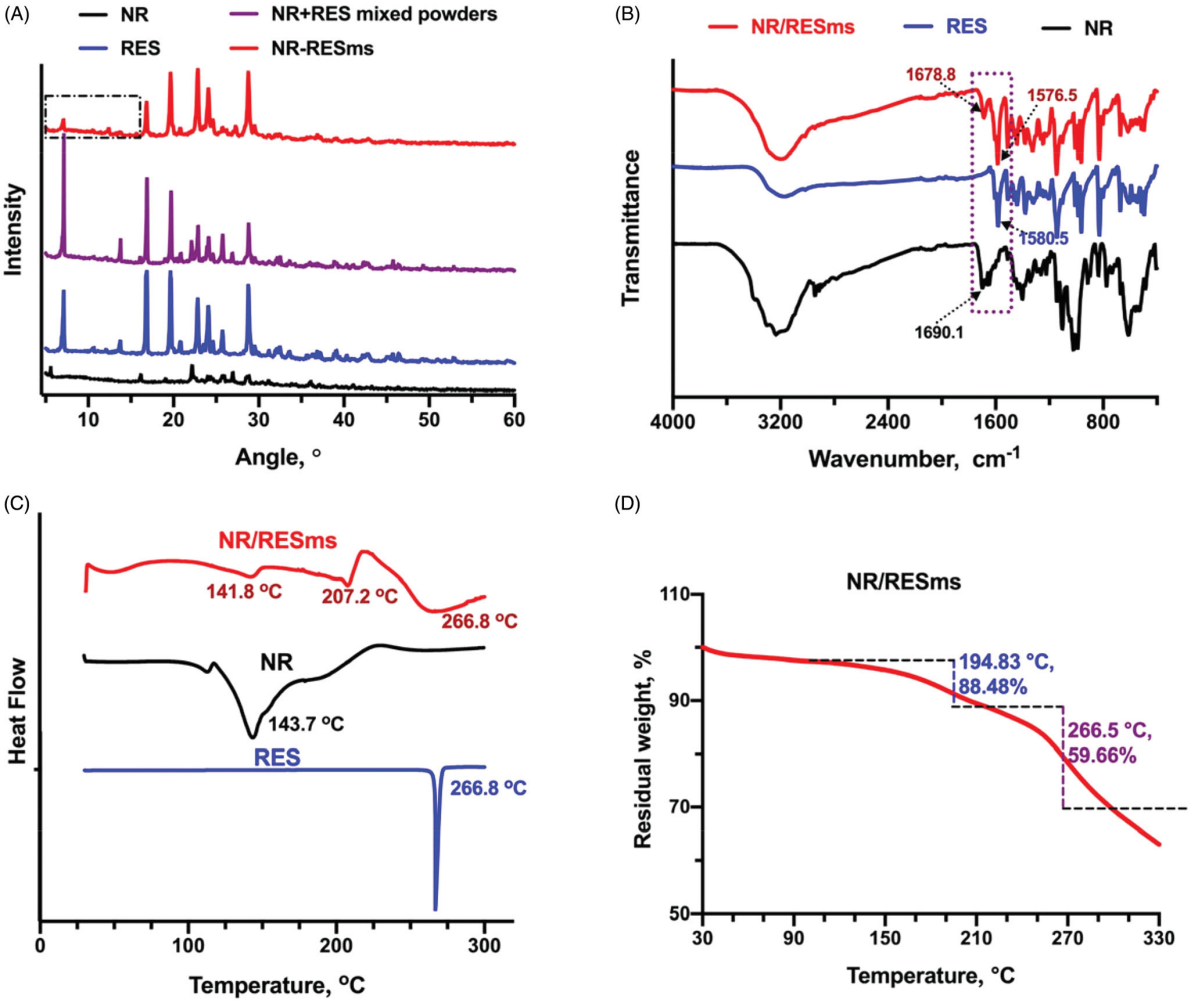
The characterization for corresponding samples: (A) XRD, (B) FTIR spectroscopy, (C) DSC; (D) the TG curve of NR/RESms.

The NR/RESms were further characterized by XRD, FT-IR, and DSC and TG. XRD results provide crystallographic information of the NR/RESms (Figure 2(A)). The crystallinity of pure RES was characterized by the strong diffraction peaks presented from 7.25 to 30 degrees. The weak diffraction peak for NR was observed because of its hydrophilic property. For the mixture of RES and NR, typic diffraction peaks were also observed. After nano spray-drying, weak diffraction peaks appeared at the corresponding point, indicating the crystallization of RES was inhibited on the surface of the NR/RESms. The absence of specific diffraction peaks in the small theta value degrees of NR/RESms suggested that an amorphous solid dispersion was constructed by the assembled NR and RES molecules.

The FT-IR spectra of NR, RES and NR/RESms were shown in Figure 2(B). The wide peaks about 3200–3400 cm^−1^ belonged to the hydroxyl groups in both NR and RES molecules. The vibrational bonds at approximately 1680 cm^−1^ are caused by the vibration of amide in the nicotinamide structure of NR molecule, and the peak at ∼1580 cm^−1^ can be attributed to the trans alkene structure of RES. The infrared spectrum for NR/RESms maintained the specific peaks as in that of pure NR and RES, which indicated the interactions by physical mixture after nano spray-drying.

The DSC thermograms of the pure NR, RES and NR/RESms were shown in Figure 2(C). The thermogram of crystallized RES exhibited a sharp endothermic peak at 266.8 °C, and a wide peak at 143.7 °C was displayed in the thermogram of hydrophilic compound NR. These results indicated that the structure of the microspheres belongs to the molecular mixture of NR and RES.

### 3.3. Release of NR from NR/RESms formulations *in vitro*

**Figure 3.**
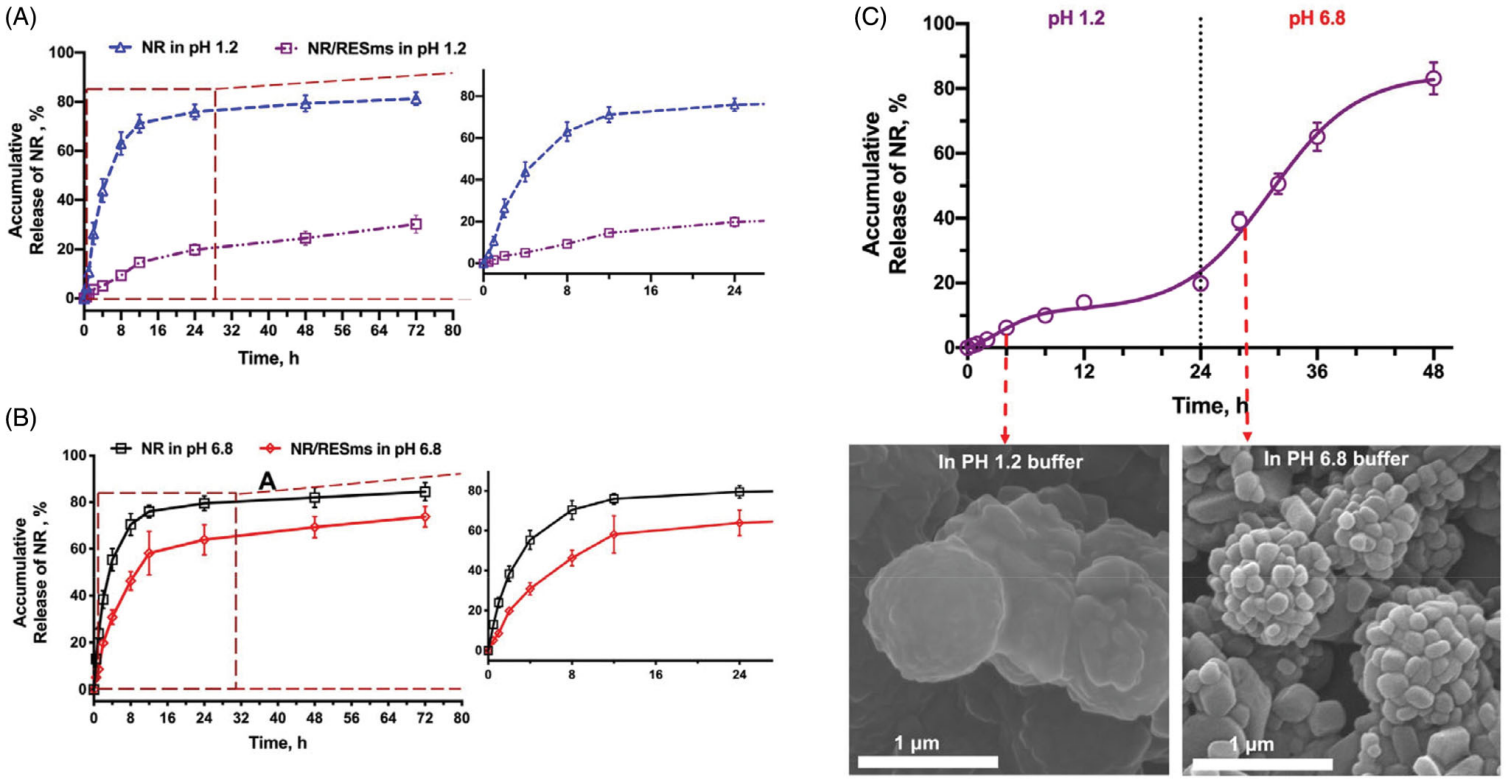
The cumulative release profiles for NR and NR/RESms using 0.2% (w/v) of alginate solution (A) in pH 1.2 buffer (0.1 M HCl); (B) in pH 6.8 buffer (phosphate buffer); (C) Release behavior of NR/RESms in alternative buffers and the micromorphology of NR/RESms in pH 1.2 and pH 6.8 buffer, respectively.

Considering that sodium alginate could form an insoluble hydrogel at acidic condition, we employed it as the vehicle for oral administration of NR/RESms (Pham & Tiyaboonchai, 2020). Before in vivo administration, drug release behavior was investigated in vitro. Figure 3(A,B) presented the release profiles of NR from NR/RESms/alginate formulation in 0.1 M HCl (pH 1.2) and phosphate buffer solution (pH 6.8), respectively. NR exhibited excellent aqueous solubility at pH 1.2, even in the 0.2% of sodium alginate solution, resulting to a release of > 60% in 8 hours (Figure 3(A)). We thought it was probably due to the diffusion effect of small molecule in medium. By contrast, the release of NR was relatively slow in the NR/RESms/alginate formulation at the gastric acid-mimic condition (<15% in 8 hours). When the pH increased to 6.8, a sustained release of NR from NR/RESms/alginate suspension was observed (accumulated NR reach to 46% within the initial 8 h), and the accumulative release of NR exceeded 60% within 24 hours (Figure 3(B)). Consistently, in alterative release experiment, a rapid drug release was also detected after the releasing buffer was switched from 0.1 M HCl to phosphate buffer solution (Figure 3(C)). Upon exposure to the gastric acidic-mimic environment (pH 1.2) for 4 hours, an insoluble sodium alginate hydrogel skeleton was formed on the surface of NR/RESms, as confirmed by SEM images (Figure 3(C)). While in the neutral or enteric environment, the alginic acid converted back to water-soluble alginate, and corrosions of microspheres were observed, which might liberate NR/RESms and allow a sustained release of NR from the microspheres (Figure 3(C)).

### 3.4. Pharmacokinetic analysis of NR/RESms in mice

**Figure 4.**
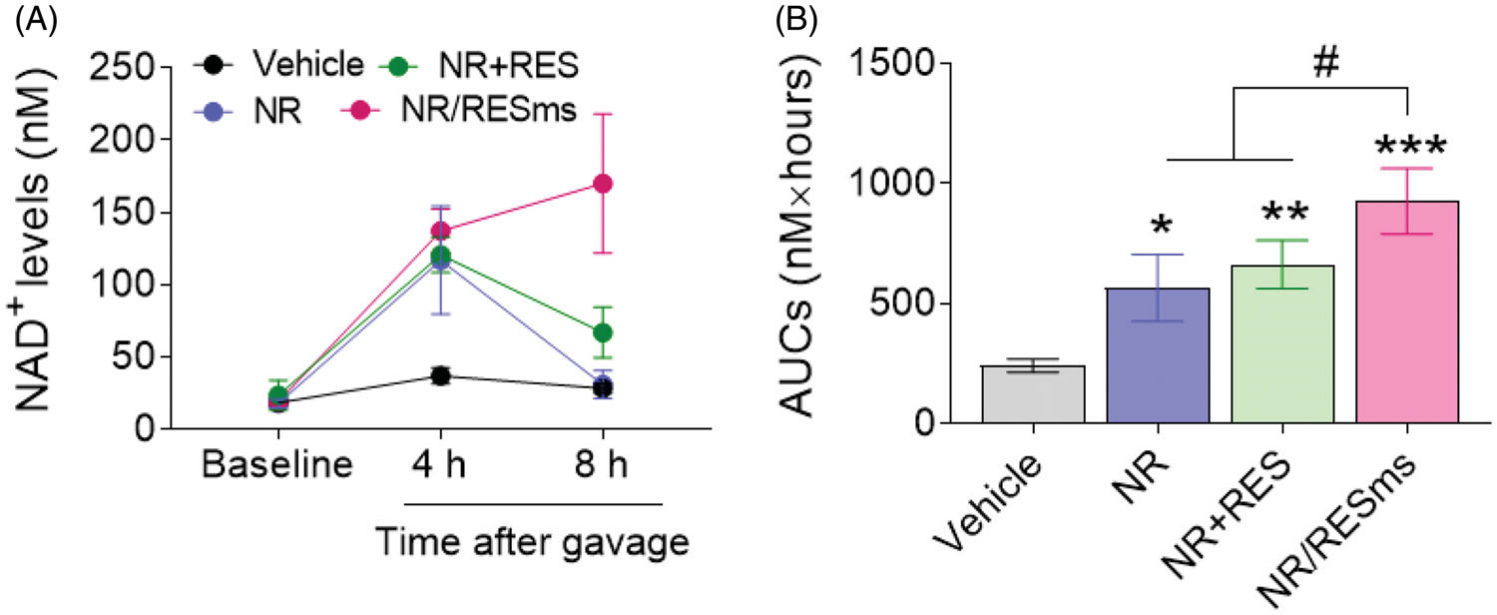
The time course of plasma concentrations of NAD^+^ after oral administration of vehicle, NR, NR + RES or NR/RESms (A) and the area under curves (AUCs) (B) (*n* = 5 per group). Data are shown as mean ± SD. *p < 0.05, **p < 0.01 and ***p < 0.001 versus Vehicle, #*p* < 0.05 vs NR/RESms.

To test whether NR/RESms is a potent NAD^+^ booster in vivo, we treated the mice with vehicle, NR, NR + RES mixture, or NR/RESms by oral gavage under a dosage of 100 mgNR/kg and analyzed the levels of NAD^+^ in serum. There was no difference in serum levels of NAD^+^ at baseline among groups. In the NR group, the plasma NAD^+^ level was increased upon NR gavage at 4 hours but declined to physiological level at 8 hours (Figure 4(A)). This decline was slightly improved in the NR + RES group, suggesting little synergetic effect on NAD^+^ elevation for a physical mixture of NR + RES (Figure 4(A)). By contrast, in the NR/RESms group, the plasma NAD^+^ level was increased at 4 hours after gavage, and this elevation persisted at 8 hours (Figure 4(A)). As a result, the abundance of NAD^+^ in circulation estimated by the area under curves (AUCs) was increased by 284% by NR/RESms during the 8 hours period after gavage as compared to the vehicle-treated mice, which was significantly higher than the NR and NR + RES groups. These results suggested that microspheres delay NR release and prolong the benefit in NAD^+^ boosting (Figure 4(B)).

**Figure 5.**
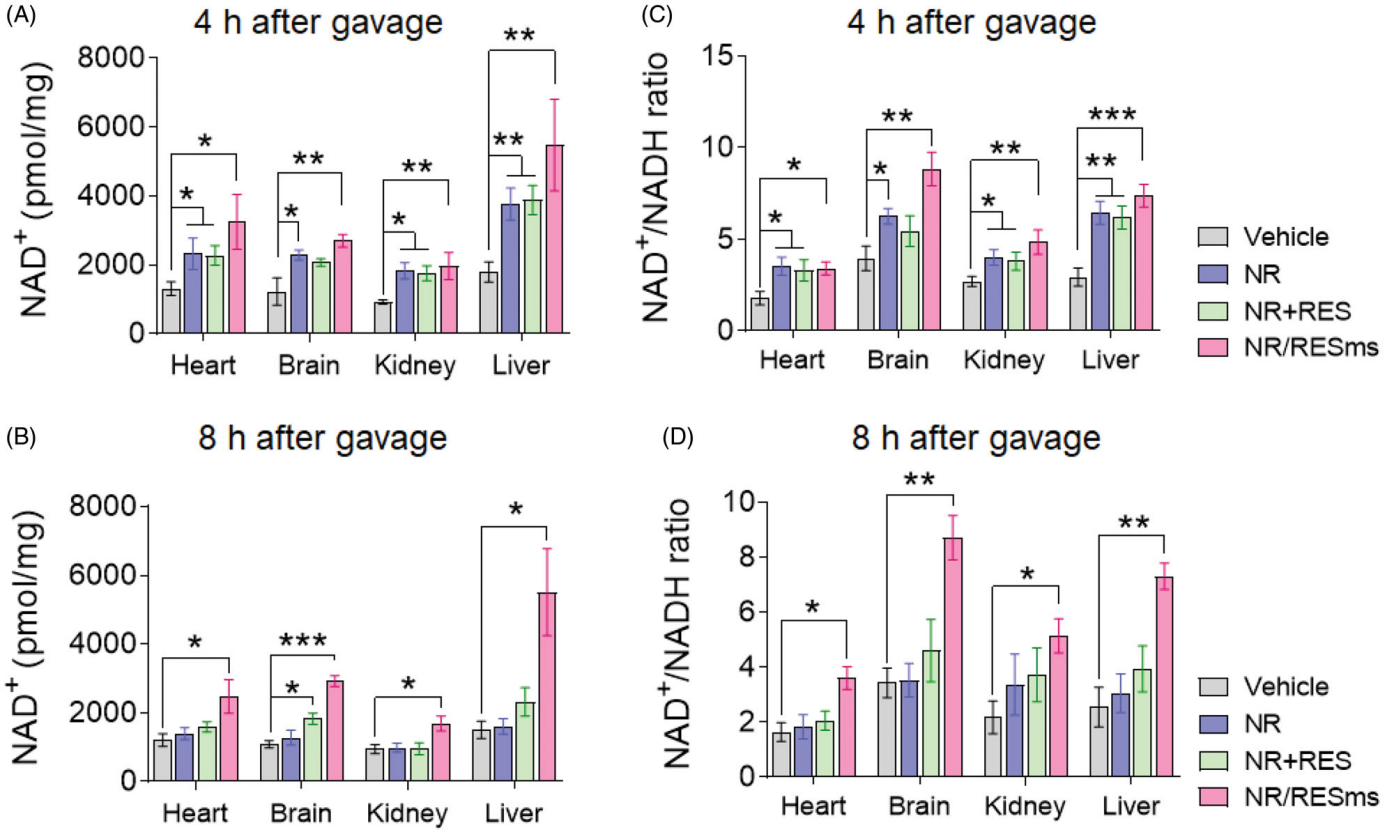
The NAD^+^ levels in heart, brain, kidney and liver at 4 (A) and 8 hours (B) after oral administration of vehicle, NR, NR + RES or NR/RESms (*n* = 4-6 per group). The NAD^+^/NADH ratio in heart, brain, kidney and liver at 4 (C) and 8 hours (D) after oral administration of vehicle, NR, NR + RES or NR/RESms (*n* = 3 per group). Data are shown as mean ± SD. *p < 0.05, **p < 0.01 and ***p < 0.001 for indicated comparisons.

To gain more insight into the pharmacokinetic characteristic of NR/RESms, we measured the NAD^+^ level and NAD^+^/NADH ratio in multiple organs. As compared to the vehicle group, the content of NAD^+^ was increased by 147% in heart, 120% in brain, 112% in kidney and 205% in liver after 4 hours of NR/RESms gavage (Figure 5(A)). Consistent with the finding in plasma NAD^+^ level, this elevation was also observed at 8 hours after NR/RESms gavage (Figure 5(B)). Interestingly, along with the increased NAD^+^ amount, the NAD^+^/NADH ratio was also upregulated, suggesting that the energetic status in the targeted organ was improved as well (Figure 5(C–D)). In the NR and NR + RES groups, increases of NAD^+^ abundance and NAD^+^/NADH ratio were only observed at 4 hours after gavage, which were absent at 8 hours (Figure 5(A–D)). Together, these results clearly indicated that NR/RESms, with a prolonged-releasing feature, improved NAD^+^ bioavailability in both circulation and multiple organs.

### 3.5. Toxicity investigation after oral administration of NR/RESms

**Figure 6.**
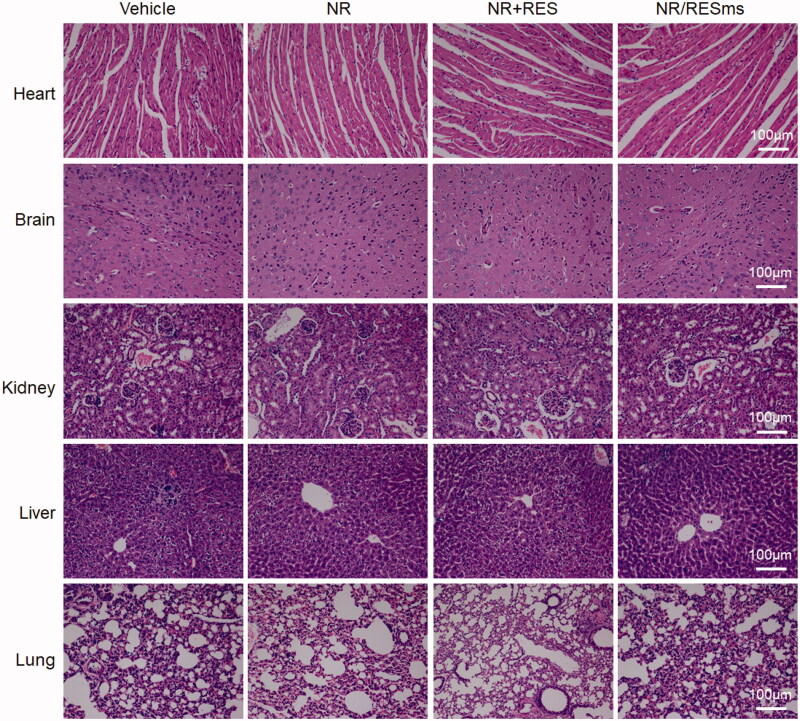
H&E staining of mouse organ slices (heart, brain, kidney, liver, and lung) after 8 hours of oral administration of vehicle, NR, NR + RES or NR/RESms. Images are representative of 3 mice in each group. Scale bar: 100 μm.

The in vivo toxicity analysis was performed in mice after oral gavage of vehicle, NR, NR + RES and NR/RESms at a dosage of 100 mgNR/kg. Among the 4 groups, none of the mice died, and we did not find evidence of uncomfortable in mice during the 8 hours of observation. The heart, brain, kidney, liver, and lung were stained with H&E and the representative images are shown in the Figure 6. No histopathological changes were observed in the NR/RESms group, as compared with the vehicle, NR and NR + RES groups. Therefore, the data suggested that NR/RESms has little toxicity in these organs.

### 3.6. Oral administration of NR/RESms attenuated cardiac I/R injury

**Figure 7.**
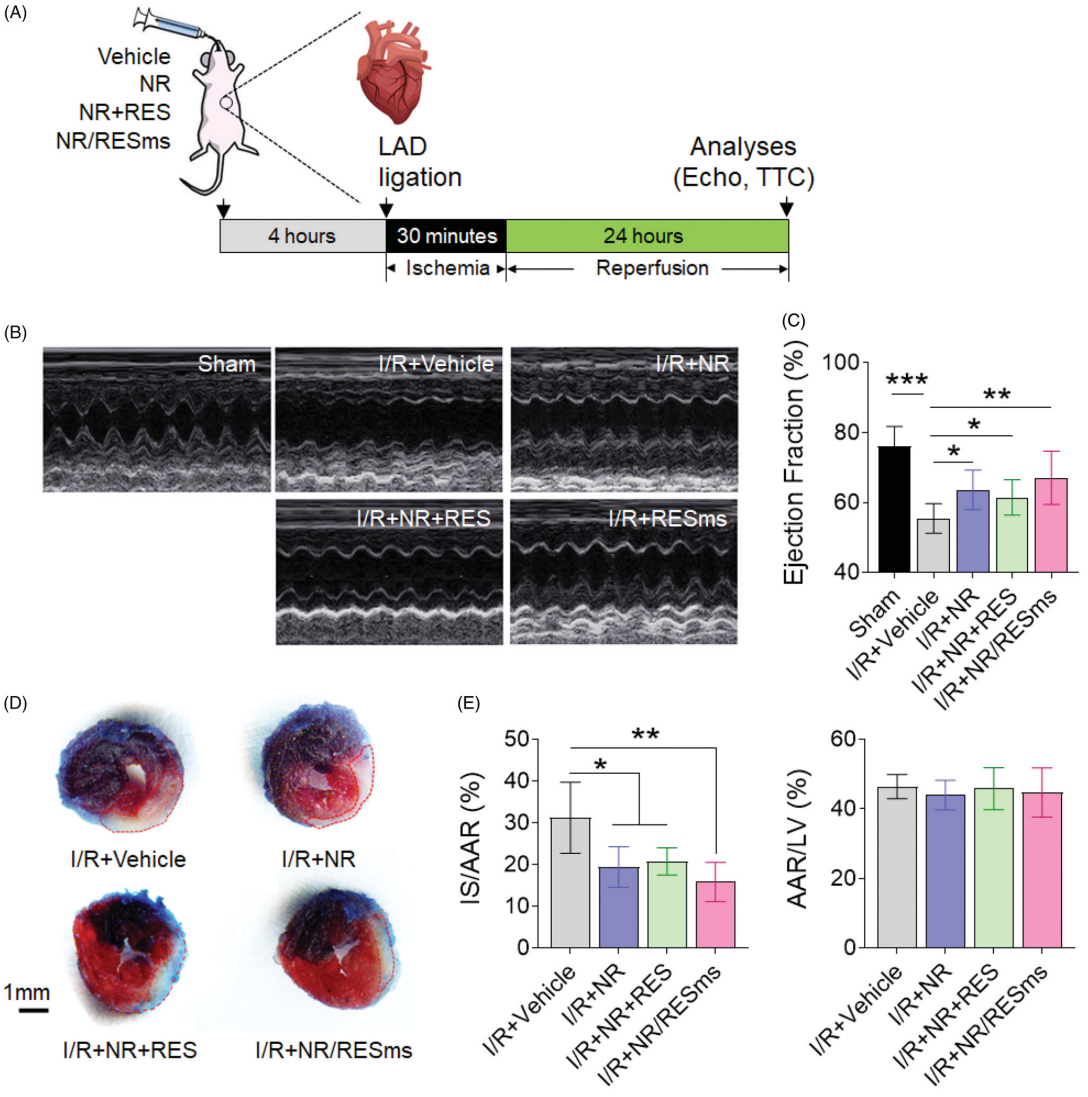
(A) Schematic representation of the experimental protocol for cardiac I/R injury; (B) Representative M-mode ultrasound cardiogram images after myocardial I/R injury or Sham operation in mice with vehicle, NR, NR + RES or NR/RESms treatment; (C). The ejection fraction as determined by echocardiography (*n* = 5 per group); (D). Representative photographs of Evans blue and tetrazolium chloride (TTC) double-stained heart sections from mice treated with vehicle, NR, NR + RES or NR/RESms and subjected to cardiac I/R injury. Blue represents unaffected, viable tissue; white represents infarct area; red + white represents area at risk (AAR). Scale bar, 1 mm; (E). Infarct size relative to AAR (IS/AAR) and AAR relative to left ventricle (AAR/LV) were quantified (*n* = 5 per group). Data are shown as mean ± SD. *p < 0.05, **p < 0.01 and ***p < 0.001 for indicated comparisons.

To test whether improving NAD^+^ abundance protects organ against acute stress, we performed cardiac LAD ligation surgery on mice received oral delivery of vehicle, NR, NR + RES or NR/RESms (Figure 7(A)). After 30 minutes of ischemia and 24 hours of reperfusion, the I/R + Vehicle group mice exhibited significantly reduced EF as compared to the Sham mice, which was markedly improved by administration of NR, NR + RES or NR/RESms, suggesting that I/R-induced functional decline was ameliorated by enhancing NAD^+^ availability (Figure 7(B–C)). Moreover, the TTC staining data showed that I/R induced an ∼30% of infarct size in the AAR, which was reduced to ∼19% by NR, 21% by NR + RES and ∼15% by NR/RESms treatment (Figure 7(D–E)). Notably, in both measurements, we observed a trend of further increase of EF and decrease of infarct size in the I/R + NR/RESms group in comparison with the I/R + NR and I/R + NR + RES groups, even though they did not reach statistical significance. Taken together, our data suggested that NR/RESms is a promising NAD^+^ booster, which could mitigate acute cardiac I/R injury.

## 4. Conclusion

In this study, we reported the nanocrystal self-assembled dual-drug microspheres fabricated by Nano Spray Dryer for oral delivery. Using physically mixed NR and RES powder as control, the morphology, chemical structure, and crystallization of the microspheres were studied, which confirmed the nanocrystal structure of NR/RESms. Due to the good biocompatibility and pH sensitivity, sodium alginate solution was employed as the vehicle for oral administration of NR/RESms. The NR/RESms/alginate formulation significantly increased NAD^+^ levels in mouse serum and multiple organs after oral administration, suggesting an improved NAD^+^ bioavailability. Furthermore, with no adverse effect on major organs, oral delivery of the NR/RESms protected heart against acute I/R injury. In conclusion, we fabricated a carrier-free dual-drug NR/RESms, and demonstrated it as an oral administrated NAD^+^ booster to attenuate cardiac I/R injury in mice.
